# Individual drivers and barriers to adoption of disease control and welfare practices in dairy and beef cattle production: a scoping review

**DOI:** 10.3389/fvets.2023.1104754

**Published:** 2023-07-06

**Authors:** Marrissa S. Buchan, Guillaume Lhermie, Sanjaya Mijar, Ed Pajor, Karin Orsel

**Affiliations:** ^1^Department of Production Animal Health, Faculty of Veterinary Medicine, University of Calgary, Calgary, AB, Canada; ^2^The Simpson Centre for Agricultural and Food Policy, The School of Public Policy, University of Calgary, Calgary, AB, Canada

**Keywords:** beef, dairy, farmer attitudes, disease prevention, biosecurity, infection control, health management

## Abstract

The implementation of disease control and welfare practices is an essential part of limiting disease exposure in livestock, however successful adoption of these practices seem to be low in both the beef and dairy cattle industries. The main objectives of this scoping review were to characterize literature published exploring beef and dairy cattle producers’ perceptions on the implementation of various disease control and welfare practices, identify major themes of drivers and barriers that influence producers’ adoption of these practices, and identify current gaps in knowledge. A total of 2,486 articles were obtained from two database literature searches and screened, from which 48 articles published between 2010 and 2021 were deemed eligible and charted. Europe was the most common region for articles (58%). A majority of articles focused solely on dairy producers (52%). A wide range of barriers and drivers which were categorized into 4 and 5 key themes, respectively. The most commonly mentioned driver was animal health, welfare, and safety, while the most common barrier was costs. Potential gaps in literature were identified, including the underrepresentation of beef producer perceptions relative to dairy producers in current literature.

## 1. Introduction

Many endemic livestock diseases are commonly classified as production-limiting diseases due to their costly economic impact on the production animal industry. In addition to economic losses, livestock diseases can also have significant impact on animal welfare and food safety, and lead to disruptions in the food supply chain (1). Furthermore, while promoting animal welfare is essential to mitigating animal suffering and improving quality of life, it can also lead to increased livestock productivity, food safety, and product quality, and is therefore beneficial to not only animals, but to producers and consumers as well (2).

Due to the complexity of these issues, many governments and commodity groups have implemented either voluntary or mandatory disease prevention programs and promote biosecurity to manage risks posed by livestock diseases, where prevention focuses on risk of introduction and control on risk of transmission. For example, the World Organization for Animal Health refers to biosecurity as “a set of management and physical measures designed to reduce the risk of introduction, establishment and spread of animal diseases, infections or infestations to, from and within an animal population” (2).

European countries have implemented quite a few control programs, many of which are compulsory, targeted toward cattle diseases, such as enzootic bovine leukosis, bluetongue, and paratuberculosis (3). In contrast, countries in North America seem to have fewer disease control programs in place, but have still implemented some voluntary programs, such as the Canadian Johne’s Disease Initiative and the US Voluntary Bovine Johne’s Disease Control Program (4, 5). These programs are essential, not only to prevent the spread of foreign animal diseases, but also to minimize the risk of endemic diseases as well (6).

Despite the economic impact and the importance of biosecurity and welfare practices in preventing and reducing the spread of diseases, the successful implementation of disease control and welfare practices seem to be low in both the beef and dairy cattle industries, in both Europe (7–9), Australasia (10, 11) and North America (12, 13). Financial factors are commonly assumed to be the major barrier to increasing the adoption of these practices, however compliance, complexity of responsibilities and limited knowledge about disease control are also commonly mentioned. In the recent years, however, researchers’ investigating disease control have placed more emphasis on exploring how stakeholder perceptions account for the disconnect in the adoption of scientific proven disease control and welfare practices. Indeed, understanding the drivers and barriers that influence producers’ perceptions toward various disease control and welfare practices may be essential to understand how to promote increased adoption of these practices, as knowledge is not necessarily the sole driver of changes. Therefore, the main objectives of this scoping review were to: (i) characterize the current literature published on producers’ perceptions surrounding various disease control and welfare practices, (ii) identify the prevailing themes of drivers and barriers and other factors that influence producers’ implementation and adoption of these practices, and (iii) unveil themes requiring further investigation.

## 2. Materials and methods

This scoping review was conducted following the methodological framework outlined by Arksey and O’Malley (14) and the Preferred Reporting Items for Systematic Reviews and Meta-Analyses Extension for Scoping Reviews (PRISMA-ScR) reporting guidelines (15).

### 2.1. Eligibility criteria

The studies of interest included primary research articles which described the drivers and barriers that cattle producers perceived regarding the implementation of various disease control and welfare practices. Any practice aimed at controlling or preventing disease was considered, including, but not limited to, vaccinations, biosecurity measures, and participation in disease control programs. Articles were required to be published in English and identify cattle producers (those that are in decision making capacity) as the population of interest in the title or abstract. Articles that had multiple different stakeholders as participants, such as veterinarians or other livestock producers, were still considered as long as cattle producers were one of the included stakeholders and there were clear indications to which perceptions were reported by cattle producers. Eligible articles were limited by geography to include North America, Europe, Australia, and New Zealand, as these geographic regions were considered economically similar in terms of gross national income (GNI) *per capita* (16).

### 2.2. Search and study selection

Literature searches were performed by the primary reviewer (MB) using 2 scientific databases to compile relevant research articles: CAB Abstracts and Web of Science. Searches were conducted on June 30th, 2022. Filters for language (English) and publication type (academic journal) were applied to database searches. Search criteria were defined using 4 concepts which were combined using the Boolean search operator “AND” (Table 1). Within each concept, terms were combined using the Boolean search operator “OR.”

**Table 1:** Boolean search term details used for literature searches conducted in CAB Abstracts and Web of Science databases.

Line	Search terms
1	Rancher* OR Producer* OR Farmer*
2	Calf OR Calves OR Cow* OR Cattle OR Beef OR Dairy
3	Attitude* OR Perception* OR Belief* OR Opinion* OR Influence* OR Behavio?r* OR Decision making
4	Animal welfare OR “Disease control” OR “Disease prevention” OR “Farm management” OR “Control programs” OR “Health management”

The articles retrieved from database searches were first downloaded and imported into Zotero reference manager software (Roy Rosenzweig Center for History and New Media, Fairfax, Virginia, United States). Articles were then imported from Zotero into Covidence systematic review software (Veritas Health Innovation, Melbourne, Australia) to aid in data management. Any duplicate articles were removed by Covidence software prior to screening. Two reviewers (MB, SM) screened all articles independently. All articles were first analyzed for relevance and eligibility based on title and abstract, then a full text screening of all approved papers was conducted by the lead author. Any conflicts during screening were resolved by consensus or a third reviewer (KO).

### 2.3. Data charting process

Data from each article that was determined relevant after the completion of the full-text screening process were charted individually using Covidence. Data charted included general study characteristics (study location, year of publication), population characteristics (cattle producer population, number of participants), study design (study methods, primary aim of study), and relevant outcomes (drivers and barriers identified) (Table 2). Multiple key themes of drivers and barriers were identified within each of the included articles through an inductive approach. This was an iterative process which was conducted throughout the data charting process based on subcategories which were identified in the literature.

**Table 2:** Description of data items obtained during charting of relevant articles.

Variable	Description of items
Study location	Region or country in which the study was conducted
Year of publication	Year the study was published
Study methods	Qualitative or mixed methodology used in the study (e.g., semi-structured interviews, survey, questionnaire, focus groups)
Cattle producer population	Specific population of cattle producers’ that participated in the study (e.g., beef, dairy, other)
Number of participants	Number of cattle producers’ that participated in the study
Primary aim of study	Disease control or welfare practice that was explored in the study
Perceived drivers and barriers identified	Factors that producers reported which motivated or deterred the implementation of a practice

## 3. Results

### 3.1. Selection of sources of evidence

**Figure 1:**
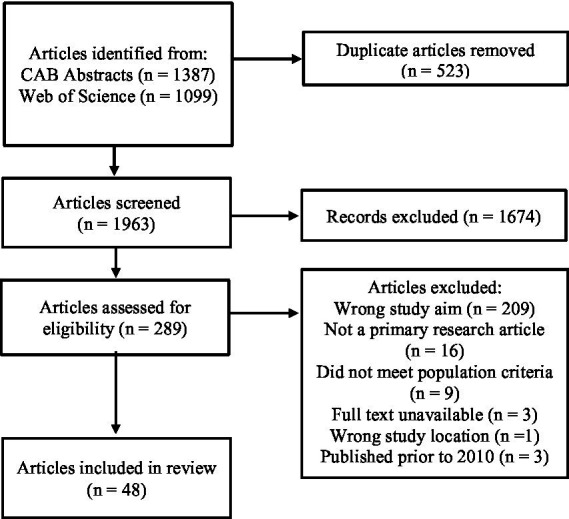
PRISMA flow diagram illustrating the database literature search, screening, and data selection process.

A total of 2,486 articles were obtained from the database literature searches of CAB Abstracts (*n* = 1,387) and Web of Science (*n* = 1,099). Once duplicate articles were removed (*n* = 523), 1,963 articles were screened by title and abstract, in which 1,674 articles were excluded. The remaining 289 articles underwent full text screening, in which 238 articles were deemed irrelevant. In total, 51 articles were deemed eligible for data charting based on full text screening. However, only 3 articles published prior to 2010 were deemed eligible. Due to the insufficient number of articles to accurately represent producer perceptions prior to 2010, these 3 articles were excluded, leaving a total of 48 articles (Figure 1).

### 3.2. Characteristics of sources of evidence

Europe was the most common region where included studies were conducted (9 from the United Kingdom, 4 from Ireland, 3 each from the Netherlands, Germany, and Belgium, 2 from Spain, and 1 each from Switzerland, Sweden, France, and Denmark). Remaining included articles were studies conducted in North America (14 from Canada and 3 from the United States), Australia and New Zealand (1 and 2 studies, respectively). The included articles had a range of publication dates from 2010 to 2022. Between 2010 and 2019, 30 articles were published, while the remaining 18 articles were published between 2020 and 2022. In terms of population of interest, a majority studies included only dairy producers (52%, *n* = 25), followed by studies that included both dairy and beef producers (17%, *n* = 8), and studies that included only beef producers (15%, *n* = 7). The remaining studies involved multiple livestock stakeholders with 5 studies involving dairy producers and veterinarians, and 1 of each study involving dairy, sheep, and goat producers, and hobby holders; dairy, beef, and sheep producers; and dairy and beef producers, producers breeding cattle for bullfighting, and veterinarians.

In terms of the research methods used to collect data in included articles, 19 studies used interviews, 18 studies reported using questionnaires, 6 studies used focus groups, 2 studies used both interviews and questionnaires, 2 studies used both focus groups and questionnaires, and 1 study used both interviews and focus groups. A variety of general management practices, diseases of interest, and welfare practices, were investigated and are characterized by study in Table 3.

**Table 3:** Data charting of eligible articles (*n* = 48).

Author, year	Study location	Aim of study	Generic management	Specific disease targeted	Specific welfare practice	Methods	Cattle population	Sample size
(17)	Ireland	HACCP-based mastitis control programs	–	Mastitis	–	Semi-structured interviews	Dairy	6
(18)	UK	Biosecurity practices	Biosecurity	–	–	Semi-structured interviews	Dairy	25
(19)	United States	Herd health management, disease control, vaccination, and antimicrobial therapy practices	Biosecurity, vaccination, antimicrobial use	–	–	Semi-structured interviews	Dairy	23
(20)	Spain	Spanish bTB eradication programme	–	Bovine tuberculosis	–	Semi-structured interviews	Beef; Dairy	21
(21)	Canada	Antimicrobial use and resistance	Antimicrobial use	–	–	Focus groups	Dairy	42
(22)	Belgium	Biosecurity practices	Biosecurity	–	–	Focus groups; Questionnaire	Beef; Dairy	8; 91
(12)	Canada	Biosecurity practices	Biosecurity	–	–	Questionnaire	Dairy	368
(23)	Ireland	Mastitis management	–	Mastitis	–	Questionnaire; Focus groups	Dairy	283; N/A
(24)	Ireland	Government-incentivized farm animal welfare programme	–	–	Weaning, disbudding, castration	Focus groups	Beef	32
(25)	USA	Antimicrobial use and alternatives	Antimicrobial use	–	–	Focus groups	Beef	39
(26)	Netherlands	BTV-8 vaccination	Vaccination	Bluetongue disease	–	Questionnaire	Dairy	707
(27)	UK	Zoonotic control program	Biosecurity	–	–	Semi-structured interviews	Beef; Dairy	43
(11)	New Zealand	Bovine viral diarrhoea control practices	–	Bovine viral diarrhoea	–	Questionnaire	Beef	71
(8)	Netherlands	Johne’s disease programme	–	Johne’s disease	–	Questionnaire	Dairy	40
(28)	UK	Lameness control	–	Lameness	–	Semi-structured interviews	Dairy	12
(29)	Netherlands	Mastitis control	–	Mastitis	–	Semi-structured interviews	Dairy	24
(30)	Germany	Veterinary advice regarding herd health	Biosecurity	–	–	Semi-structured interviews	Dairy	38
(31)	Canada	Lameness prevention and control	–	Lameness	–	Semi-structured interviews	Dairy	3
(32)	Australia	Infectious bovine keratoconjunctivitis treatments	–	Infectious bovine keratoconjunctivitis	–	Questionnaire	Beef; Dairy	1,644
(13)	Canada	Bovine leukemia virus control measures	–	Bovine leukemia	–	Focus groups	Dairy	24
(33)	UK	Lameness control	–	Lameness	–	Questionnaire	Dairy	222
(34)	UK	Bovine viral diarrhoea management	–	Bovine viral diarrhoea	–	Questionnaire	Beef; Dairy	43
(35)	Canada	Handling, weaning, and euthanasia management practices	–	–	Handling, weaning, euthanasia	Semi-structured interviews; Questionnaire	Beef	15; 94
(36)	Canada	Pain mitigation practices	–	–	Pain mitigation	Semi-structured interviews; Questionnaire	Beef	15; 94
(37)	Spain	Biosecurity practices	Biosecurity	–	–	Semi-structured interviews	Dairy	16
(38)	Canada	Cow comfort	–	–	Cow comfort	Questionnaire	Dairy	118
(39)	New Zealand	Providing cow-calf contact	Biosecurity	–	–	Semi-structured interviews	Dairy	67
(40)	Denmark	Biosecurity practices	Biosecurity	–	–	Semi-structured interviews	Dairy	16
(41)	Switzerland	Calf health management and antibiotic use	Biosecurity, antimicrobial use	–	–	Semi-structured interviews	Beef; Dairy	17
(42)	France	Digital dermatitis treatments	–	Digital dermatitis	–	Questionnaire	Dairy	65
(43)	Belgium	Biosecurity practices	Biosecurity	–	–	Questionnaire	Beef; Dairy	100
(44)	Germany	Veterinary herd health management (VHHM)	Biosecurity	–	–	Questionnaire	Dairy	216
(45)	Canada	Johne’s disease control program	–	Johne’s disease	–	Questionnaire	Dairy	224
(46)	UK	Johne’s disease control	–	Johne’s disease	–	Semi-structured interviews	Dairy	13
(47)	Canada	Johne’s disease control	–	Johne’s disease	–	Focus groups	Dairy	39
(48)	Canada	Pain control for disbudding and dehorning practices	–	–	Dehorning pain management	Semi-structured interviews	Dairy	29
(49)	Ireland	Biosecurity practices	Biosecurity	–	–	Questionnaire	Dairy	444
(50)	Canada	Outdoor access	–	–	Outdoor access	Semi-structured interviews; Focus groups	Dairy	6; 47
(51)	Canada	Voluntary Johne’s disease control program	–	Johne’s disease	–	Questionnaire	Dairy	238
(52)	Sweden	Veterinary recommendations for preventive measures	Biosecurity	–	–	Semi-structured interviews	Beef; Dairy	169
(53)	UK	E. coliO157 control measures	–	E. coliO157	–	Questionnaire	Beef; Dairy	405
(54)	UK	Lameness treatment and control	–	Lameness	–	Semi-structured interviews	Beef	21
(55)	UK	Lameness treatment and control	–	Lameness	–	Questionnaire	Beef	532
(56)	Belgium	Gastrointestinal nematode (GIN) control	–	Gastrointestinal nematodes	–	Semi-structured interviews	Dairy	22
(57)	United States	Antibiotic use and resistance	Antimicrobial use	–	–	Semi-structured interviews	Dairy	21
(58)	Canada	Calf care practices	–	–	Neonatal calf care	Focus groups	Dairy	23
(59)	Canada	Disbudding and dehorning practices	–	–	Dehorning pain management	Questionnaire	Dairy	165
(60)	Germany	Johne’s disease control and voluntary control programme	–	Johne’s disease	–	Questionnaire	Beef; Dairy	225

### 3.3. Drivers of adoption

**Figure 2:**
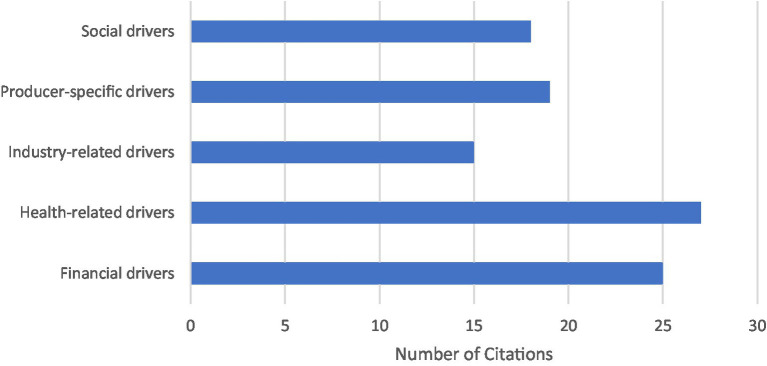
Overall number of perceived driver key themes identified in included studies (*n* = 35). Multiple themes could be identified in one study.

Out of the 48 articles included in this review, 73% discussed factors that producers perceived as drivers for implementation of various disease control and welfare practices (*n* = 35). A majority of articles which discussed these drivers were focused only on dairy producers (*n* = 26). Among the remaining articles, 4 focused only on beef producers and 5 included both beef and dairy producers. Five key themes of perceived drivers of disease control and welfare implementation were identified from included studies: health-related drivers, financial drivers, producer-specific drivers, social drivers, and industry-related drivers (Figure 2).

Health-related drivers were the most commonly identified within included articles (*n* = 27). The subcategories included promoting the animal health, welfare, and safety (*n* = 24), disease prevention (*n* = 5), ensuring staff health and safety (*n* = 4), and producers concerns regarding public health (*n* = 3). The second most identified driver within included articles were financial drivers (*n* = 25). This theme included the affordability of a practice (*n* = 1), financial gains (*n* = 10) and avoiding losses (*n* = 2), impacts on productivity (*n* = 6) and production losses (*n* = 3), savings through future cost reductions (*n* = 4), and the implemented financial incentives (*n* = 8) and penalties (*n* = 4). A total of 19 studies identified factors which were considered specific to each producers’ experiences and personal beliefs and values, labeled producer-specific drivers. This included the subcategories of intrinsic motivations (*n* = 6), which included feelings such as satisfaction or pride, personal experiences (*n* = 3), producers’ perceived sense of responsibility (*n* = 3), producers’ perceived risk of a disease or need for implementation of a practice (*n* = 3), and the perceived effectiveness (*n* = 2), feasibility (*n* = 3), and convenience (*n* = 6) of a practice. Social drivers were identified in 18 articles, which included the following subcategories: veterinarian advice (*n* = 12), public and consumer perceptions (*n* = 10), and social influences such as adoption by other producers (*n* = 3). Industry-related drivers were identified in 15 articles and included the subcategories of government and industry regulations (*n* = 11), market access or demand (*n* = 4), and food safety and production quality (*n* = 3).

In terms of geography, the 15 studies which discussed drivers and were conducted North America most frequently identified health-related drivers (80%, *n* = 12), followed by financial drivers (60%, *n* = 9), producer-specific drivers (47%, *n* = 7), social drivers (47%, *n* = 7), and industry drivers (40%, *n* = 6). Similarly, the 2 studies conducted in New Zealand also identified health-related drivers (100%, *n* = 2) most frequently, whereas financial drivers, producer-specific drivers, social drivers, and industry drivers were each mentioned in 1 study (50%). In contrast, in studies conducted in Europe (*n* = 18), financial drivers were most frequently identified (83%, *n* = 15), followed by health-related drivers (72%, *n* = 13). Producer-specific drivers, social drivers, and industry drivers were identified in 11 studies (61%), 10 studies (56%), and 8 studies (44%), respectively.

A total of 22 included articles that discussed drivers were published between 2010 to 2019, and 13 articles were published between 2020 and 2022. Financial drivers were identified in 16 studies in the 2010’s (73%) and 9 studies in the 2020’s (69%). Health-related drivers were also identified in 14 studies in the 2010’s (64%) and 13 studies in the 2020’s (100%). Producer-specific drivers were identified in 12 studies in the 2010’s (55%) and 7 studies in the 2020’s (54%). Social drivers were identified in 11 studies in the 2010’s (50%) and 7 studies in the 2020’s (54%). Lastly, industry drivers were identified in 10 studies in the 2010’s (45%) and 5 studies in the 2020’s (38%).

**Figure 3:**
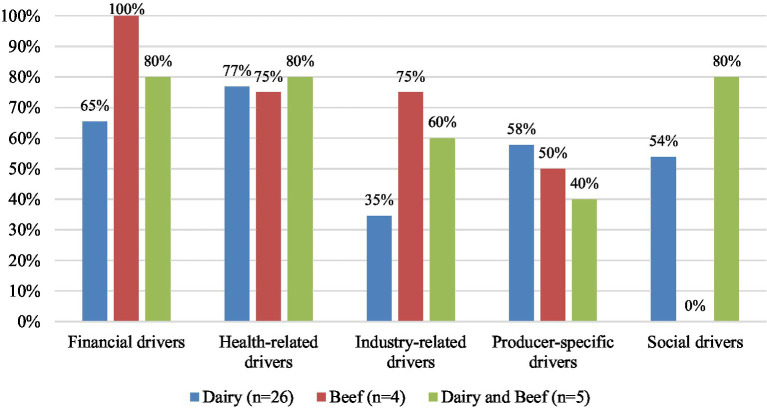
Percentage of key themes of perceived drivers identified in studies with dairy producers (*n* = 26), beef producers (*n* = 4), and both dairy and beef producers (*n* = 5). Some studies reported multiple barriers, therefore the sum of percentages for each commodity are greater than 100%.

When categorized by commodity (Figure 3), health-related drivers were identified in 80% of studies including both beef and dairy producers (*n* = 4), 77% of studies with only dairy producers (*n* = 20), and 75% of studies with only beef (*n* = 3). Financial drivers were mentioned most frequently in studies with only beef producers (100%, *n* = 4), followed by studies with both beef and dairy producers (80%, *n* = 4), and 65% of studies with only dairy (*n* = 17). Producer-specific drivers were identified in 58% of studies with only dairy producers (*n* = 15), 50% studies with only beef producers (*n* = 2), and 40% studies with both beef and dairy producers (*n* = 2). Social drivers were identified in 80% of studies with both beef and dairy (*n* = 4) and 54% of studies with only dairy (*n* = 14) but were not mentioned in any studies with only beef producers. Industry-related drivers were most commonly identified in studies with only beef producers (75%, *n* = 3), followed by studies with both beef and dairy producers (60%, *n* = 3), and studies with only dairy producers (35%, *n* = 9).

### 3.4. Barriers to adoption

**Figure 4:**
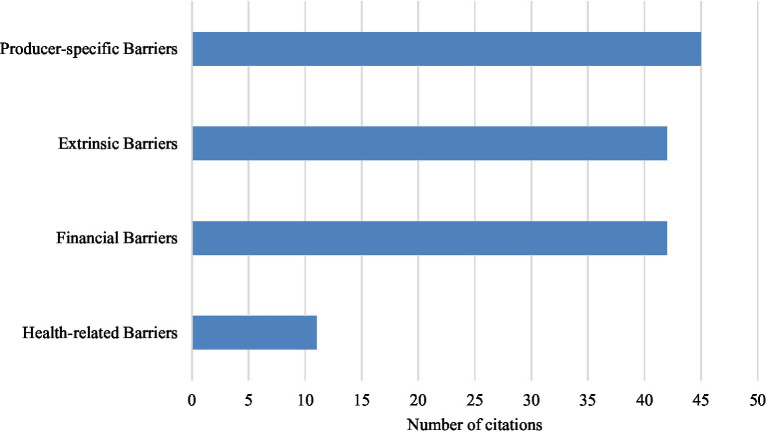
Overall number of perceived barrier key themes identified in included studies (*n* = 47). Multiple themes could be identified in one study.

In total, 98% (n = 47) of the included articles in this review discussed barriers perceived by producers which hindered their adoption of disease control and welfare practices. Of these articles, 30 studies focused only on dairy producers, 7 studies focused only on beef producers, and 10 focused on both dairy and beef producers. The following four key themes of perceived barriers to the adoption of disease control and welfare practices were identified from included studies: producer-specific barriers, extrinsic barriers, financial barriers, and health-related barriers (Figure 4).

The most identified barriers within the included articles were producer-specific barriers (*n* = 45). Producer-specific barriers included the subcategories of producers perceived risk of a disease or need for the implementation of a given practice (*n* = 25), perceived effectiveness of a practice (*n* = 23), lack of awareness or knowledge (*n* = 20), feasibility of implementing a practice (*n* = 17), lack of motivation (*n* = 5), habit or tradition (*n* = 4), and producers’ lack of sense of responsibility to adopt a given practice (*n* = 3). A total of 42 included articles identified extrinsic barriers, which was comprised external influences and included the following subcategories: time constraints (*n* = 30), labor constraints (*n* = 22), inadequate facilities or space (*n* = 23), workload and required effort (*n* = 21), weather (*n* = 4), and public and consumer perceptions (*n* = 3). Financial barriers were identified in 42 included articles. The subcategories included costs (*n* = 35), financial limitations (*n* = 5), impact on productivity (*n* = 10), lack of financial incentives (*n* = 4), market demand and supply quotas (*n* = 4), and lack of financial gains (*n* = 4). In total, 11 included articles identified health-related barriers, which included the subcategories of perceived negative impacts on animal health, welfare, and safety (*n* = 10), and staff health and safety (*n* = 3).

When categorized by geography, North American studies (*n* = 16) identified financial barriers (100%, *n* = 16) most frequently, followed by extrinsic barriers (94%, *n* = 15), producer-specific barriers (88%, *n* = 14), and health-related barriers (38%, *n* = 6). Studies conducted in Australia and New Zealand (*n* = 3) identified both financial barriers and producer-specific barriers in every article (100%, *n* = 3), followed by extrinsic barriers (67%, *n* = 2) and health-related barriers (33%, *n* = 1). The 28 studies conducted in Europe identified producer-specific barriers in every study (100%, *n* = 28), whereas extrinsic barriers, financial barriers, and health-related barriers, were identified in 25 studies (89%), 23 studies (82%), and 4 studies (14%), respectively.

In total, 29 articles included in this review which discussed barriers were published between 2010 to 2019, and 18 articles were published between 2020 and 2022. Producer-specific barriers were identified in 28 studies in the 2010’s (97%) and 17 studies in the 2020’s (94%). Financial barriers were identified in 24 studies in the 2010’s (83%) and 18 studies in the 2020’s (100%). Extrinsic barriers were identified in 28 studies in the 2010’s (97%) and 14 studies in the 2020’s (78%). Lastly, health-related barriers were identified in 5 studies in the 2010’s (17%) and 6 studies in the 2020’s (33%).

**Figure 5:**
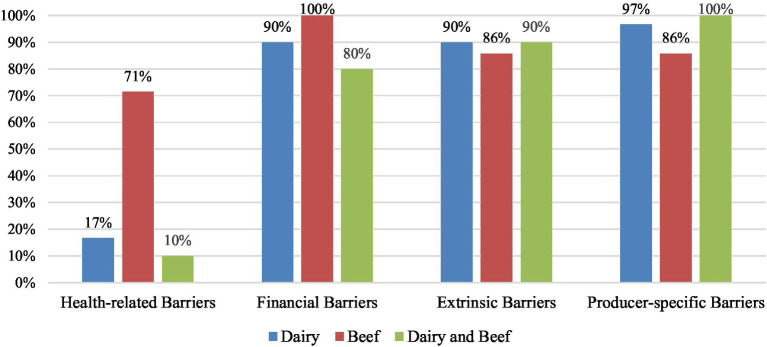
Percentage of key themes of perceived barriers identified in studies with dairy producers (*n* = 31), beef producers (*n* = 7), and both dairy and beef producers (*n* = 11). Some studies reported multiple barriers, therefore the sum of percentages for each commodity are greater than 100%.

In terms of differences in commodities (Figure 5), producer-specific barriers were most frequently identified in studies with both beef and dairy producers (100%, *n* = 10), followed by studies with only dairy producers (97%, *n* = 29), and studies with only beef producers (86%, *n* = 6). Extrinsic barriers were identified in 90% of studies with only dairy producers (*n* = 27) and both beef and dairy producers (*n* = 9), and 86% of studies with only beef (*n* = 6). Financial barriers were identified in 100% of studies with only beef (*n* = 7), 90% of studies with only dairy (*n* = 27), and 80% of studies with both beef and dairy producers (*n* = 8). Health-related barriers were mentioned in most frequently in studies with only beef producers (71%, *n* = 5), followed by studies with only dairy producers (17%, *n* = 5), and studies with both beef and dairy producers (10%, *n* = 1).

## 4. Discussion

This scoping review identified a range of barriers and drivers which dairy and beef producers perceive regarding the implementation of disease control and welfare practices. These results indicate that the factors that influence producers’ decisions regarding these practices are complex and extensive. This is an interesting finding as literature published before 2000 focuses primarily on economic drivers, and only more recent literature identifies the multifactorial complexity.

In terms of adoption, animal health, welfare and safety were the most mentioned drivers. Producers frequently spoke of their desire to promote animal health, welfare, and safety, to ensure that their animals were comfortable and had limited exposure to stressful or harmful experiences. Intrinsic motivators, such as producers’ sense of pride, responsibility, or satisfaction, were also associated with improving their livestock’s quality of life.

Producers also often associated their decision to implement a given practice with the advice received from their veterinarians. This suggests that, through developing positive relationships with their clients, veterinarians may play a key role in influencing the uptake of disease control and welfare practices. This may also be reflected in the fact that producers lack of awareness or knowledge regarding these practices was identified as a major barrier to implementation. By effectively educating their clients on the benefits and importance of these practices, especially in regard to disease prevention, veterinarians may be able to lessen this barrier for producers in the future.

In addition to a lack of awareness and knowledge, other producer-specific barriers were commonly mentioned as well. Many producers believed that their farms were not at risk to diseases and that changes to given practices were not needed. Additionally, many producers mentioned that they perceived various practices as ineffective or not feasible to implement on their farm. However, our results indicate that these producer-specific barriers alone are not responsible for limited adoption, as the costs of implementing a practice and other financial limitations, such as a lack of funds, was identified to be a frequent barrier for producers. Nevertheless, extrinsic barriers were mentioned as frequently as financial barriers, which included inadequate facilities or space to allow for adoption, time constrains, overwhelming workloads, and labor constraints, such as limited availability, lack of skilled staff, and difficulties with staff compliance. This might imply that factors which producers may perceive as out of their control are having a strong negative impact on their adoption rates. In addition to this, a majority of the studies included in this review focused solely on dairy producers’ perceptions, indicating that beef producers’ perceptions seem to be underrepresented in the literature. It is apparent in this review that there are differences between dairy and beef producers in some of the drivers and barriers that are perceived relevant. For example, the beef industry seems to be motivated by financial and industry-related drivers, and most perceived barriers are health-related, whereas dairy producers identify more producer-specific drivers.

Where dairy producers identify more producer specific drivers. Differences in barriers and drivers in the different commodity groups should inform industry initiatives for disease control strategies to be more targeted to ultimately be more effective. More focused research regarding factors which influence the adoption of disease control and welfare practices that are specific to the beef industry can assist with more broadly supported adoption. In addition to differences between commodity groups, there are also differences due to location (Europe, North America, and Australasia). This should stimulate more research to assess lessons learned from other parts of the world, as well as how differences in industry practices and management affect drivers and barriers.

The results of this review showed that barriers to implementation of these practices were more commonly investigated relative to drivers. Further investigation into the producers’ perception of the importance of improving drivers versus mitigating barriers in influencing their actions may be beneficial in determining how to better promote the implementation of disease control and welfare practices. Additionally, very few articles regarding herd-level welfare practices aiming at limiting or preventing disease were detected in our literature search, resulting in welfare being reported under health-related issues.

Some limitations of this study included possible articles that may have been missed from our review due to the exclusion of articles not written in English, that were unavailable for full text screening, or were published in countries currently excluded due to low GNI *per capita* from this review, as well as any articles that were not acquired through literature search due to possibly missing criteria and exclusion of literature not published in academic journals. Especially governmental and industry reports could harbor relevant information on disease control as related to the commodity groups in those specific countries and could have informed the results even more. However, access and quality assurances are more challenging to assess and therefore any form of gray literature was excluded from this review. Finally, as only two major databases where used, additional databases could have recovered additional papers not present in CAB Abstracts or Web of Science.

Overall, while financial factors seemed to have been the key barrier to producers’ implementation of disease control and welfare practices in the past, there are many other influential factors involved. Also, other factors besides economics are all published more recently, and therefore most likely only recently identified as important factors to successful implementation of disease prevention and control strategies. This scoping review identified and summarized many key barriers and drivers which producers perceive when making decisions regarding disease control and welfare management on their farm. This review may be useful to inform direction of future studies that aim to investigate and obtain a more comprehensive understanding of how to mitigate barriers or promote drivers acknowledging the producer specific needs in management and control strategies. Gaining a better understanding of the level of influence these factors have as well as the ways in which these factors relate may be essential to increasing the adoption rate of these practices.

## Data availability statement

The original contributions presented in the study are included in the article/supplementary material, further inquiries can be directed to the corresponding author.

## Author contributions

MB initiated the literature search and data screening and drafted the manuscript. SM did the full-text screening of articles identified through the literature search. EP and GL provided the guidance in the initial question formulation and feedback on manuscript drafting. KO supervised MB in the process and steered the writing process and approval of the final draft. All authors contributed to the article and approved the submitted version.

## Funding

MB was supported by the National Sciences and Engineering Research Council of Canada (NSERC), the Anderson-Chisholm Chair in Animal Care and welfare and the Simpson Centre for Food and Agricultural Policy, Calgary, Canada.

## Conflict of interest

The authors declare that the research was conducted in the absence of any commercial or financial relationships that could be construed as a potential conflict of interest.

## Publisher’s note

All claims expressed in this article are solely those of the authors and do not necessarily represent those of their affiliated organizations, or those of the publisher, the editors and the reviewers. Any product that may be evaluated in this article, or claim that may be made by its manufacturer, is not guaranteed or endorsed by the publisher.
